# Sarcoidosis Masquerading as Adjustment Disorder: A Case Report of Hypercalcemia-Induced Altered Sensorium

**DOI:** 10.7759/cureus.95236

**Published:** 2025-10-23

**Authors:** Keerthana Sri, Sowmya Gopalan

**Affiliations:** 1 Internal Medicine, Sri Ramachandra Institute of Higher Education and Research, Chennai, IND; 2 General Medicine, Sri Ramachandra Institute of Higher Education and Research, Chennai, IND

**Keywords:** altered sensorium, ebus, endobronchial ultrasound, non-caseating granuloma, pulmonary sarcoidosis, severe hypercalcemia

## Abstract

Sarcoidosis is a multisystem granulomatous disease that can present with nonspecific symptoms and, rarely, with severe hypercalcemia and neurological manifestations.

We report a case of a 55-year-old female with systemic hypertension, type 2 diabetes mellitus, and hypothyroidism, who presented with reduced appetite for five months, altered sensorium for three months, and difficulty rising from bed. Initial evaluation elsewhere diagnosed adjustment disorder. On further workup, she was found to have marked hypercalcemia (serum calcium = 16.7 mg/dL), renal dysfunction (creatinine = 3.7 mg/dL), and a chest X-ray showed hilar lymphadenopathy. Positron emission tomography-computed tomography revealed periportal and mediastinal lymphadenopathy. Endobronchial ultrasound-guided biopsy showed necrotizing granulomatous inflammation. Acid-fast bacillus testing was negative, and serum angiotensin-converting enzyme levels were elevated. A diagnosis of sarcoidosis was made. The patient was treated with intravenous fluids, correction of electrolyte imbalance, and corticosteroids. Clinical improvement was observed with normalization of mental status and renal function within three days. This case highlights the importance of considering sarcoidosis as the underlying cause of hypercalcemia in a patient with neurological symptoms such as depression. Early recognition and treatment can prevent long-term complications.

## Introduction

Sarcoidosis is a systemic granulomatous inflammatory disorder of unknown etiology that most commonly affects the lungs and intrathoracic lymph nodes, but may involve virtually any organ. The clinical presentation is heterogeneous and ranges from asymptomatic radiographic findings to multisystem disease with constitutional symptoms, organ dysfunction, and metabolic complications [1-4]. Hypercalcemia in sarcoidosis most commonly results from extrarenal production of 1,25-dihydroxyvitamin D (calcitriol) by activated macrophages within granulomatous tissue. Unlike renal synthesis, this extrarenal pathway is not tightly regulated by parathyroid hormone or serum calcium levels, leading to sustained overproduction of calcitriol. The consequent increase in intestinal calcium absorption predisposes patients to hypercalcemia and hypercalciuria, both of which can contribute to renal complications such as nephrolithiasis, nephrocalcinosis, and progressive renal dysfunction. In addition, elevated calcium levels may produce systemic manifestations, including fatigue, muscle weakness, and neuropsychiatric symptoms, thereby compounding the overall disease burden [4-6].

Neurological involvement in sarcoidosis, known as neurosarcoidosis, can occur through two principal mechanisms. First, direct granulomatous infiltration of the central or peripheral nervous system can involve the meninges, cranial nerves, spinal cord, or cerebral parenchyma, resulting in a wide clinical spectrum that ranges from chronic meningitis and cranial neuropathies to seizures and myelopathy. Second, neurological symptoms may arise indirectly from metabolic or toxic consequences, most notably those associated with severe hypercalcemia. Hypercalcemia-induced encephalopathy can manifest as cognitive impairment, mood disturbances, lethargy, psychosis, or, in extreme cases, coma. Thus, neurosarcoidosis reflects a complex interplay between localized granulomatous inflammation and systemic metabolic dysregulation, both of which may coexist and amplify neurological morbidity [6-9].

This case report emphasizes the importance of a high index of suspicion for sarcoidosis in patients with systemic symptoms and severe electrolyte disturbances.

## Case presentation

A 55-year-old female, residing in Andaman, presented with reduced appetite for five months and altered sensorium for three months. She also experienced progressive difficulty getting out of bed. Her medical history included systemic hypertension, type 2 diabetes mellitus, and hypothyroidism, all medically controlled. She was initially evaluated in a local hospital in May 2024, where she was diagnosed with adjustment disorder and treated with supportive measures. Her condition deteriorated over time, prompting further evaluation.

On clinical examination, the patient was drowsy, dehydrated, and responding to simple oral commands, with stable vitals. On systemic examination, no respiratory distress or other signs of infection were noted.

Neurological examination revealed the following findings: consciousness and orientation testing showed the patient was alert and oriented to time, place, and person. Higher mental functions testing showed a Mini-Mental State Examination (MMSE) score of 28/30, with intact attention, memory, comprehension, and abstract thinking, and depressed mood with psychomotor retardation.

Cranial nerves (II-XII) were intact, pupils were equal and reactive, extraocular movements were full, and no facial asymmetry or dysarthria was noted. Motor system testing revealed normal tone, power of 5/5 in all limbs, and no involuntary movements, with mild proximal fatigability noted on sustained activity. Reflexes testing showed deep tendon reflexes were present and symmetrical, with plantar reflex flexor bilaterally, and no clonus. Sensory system testing revealed pain, temperature, vibration, and proprioception intact, with no dermatomal sensory loss. Coordination testing revealed normal finger-nose and heel-knee tests, with no dysmetria or past pointing. The gait was normal. Autonomic function testing revealed no orthostatic hypotension.

**Figure 1:**
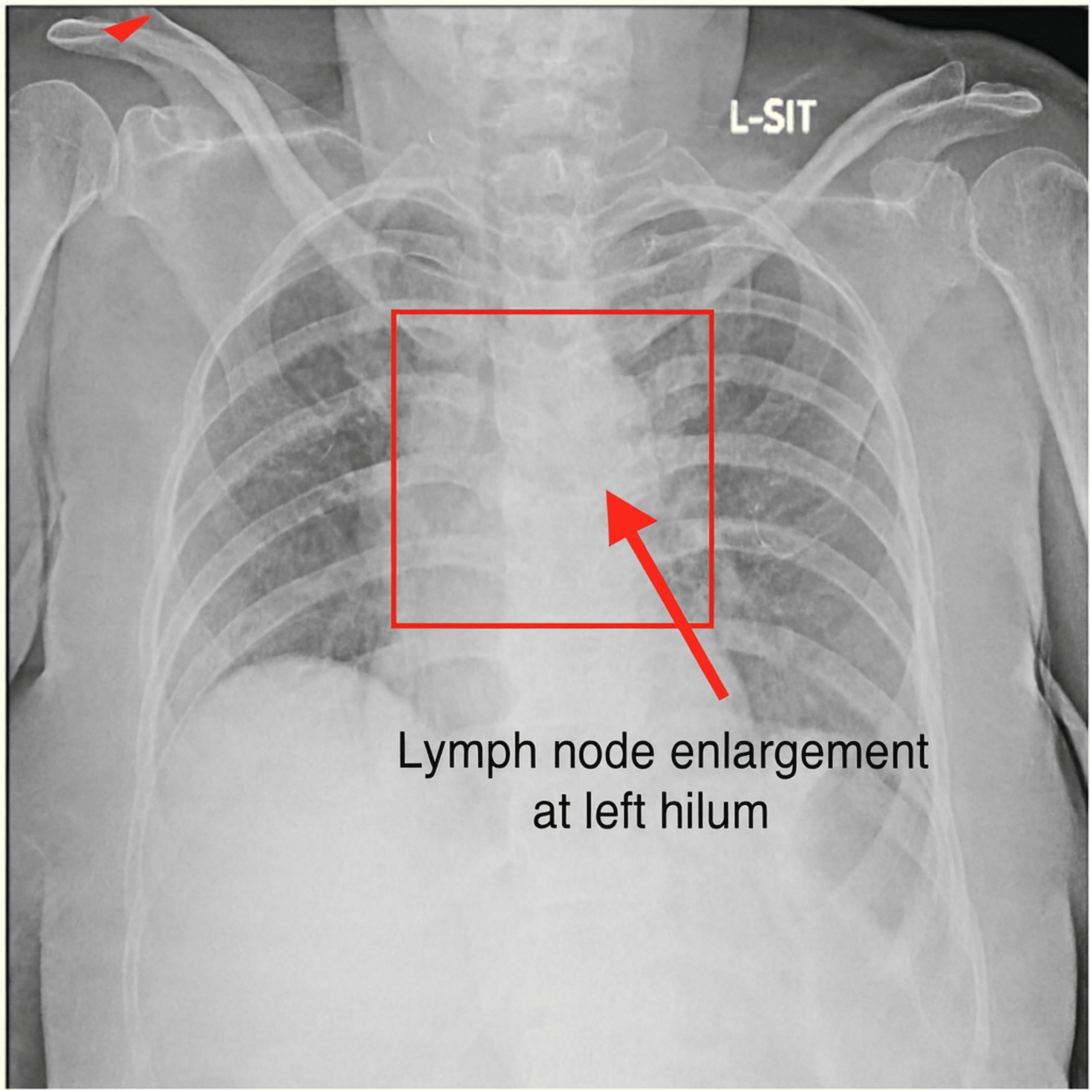
Chest X-ray (posteroanterior view) showing lymph node enlargement at the left hilum (red arrow).

**Figure 2:**
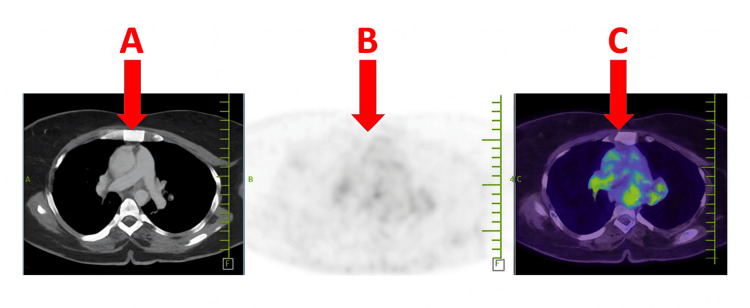
Axial PET-CT images of the thorax. (A) CT showing periportal lymph nodes. (B) PET grayscale image. (C) Fused PET-CT highlighting fluorodeoxyglucose-avid periportal lymphadenopathy (red arrows), consistent with sarcoidosis-related lymphadenopathy.

**Figure 3:**
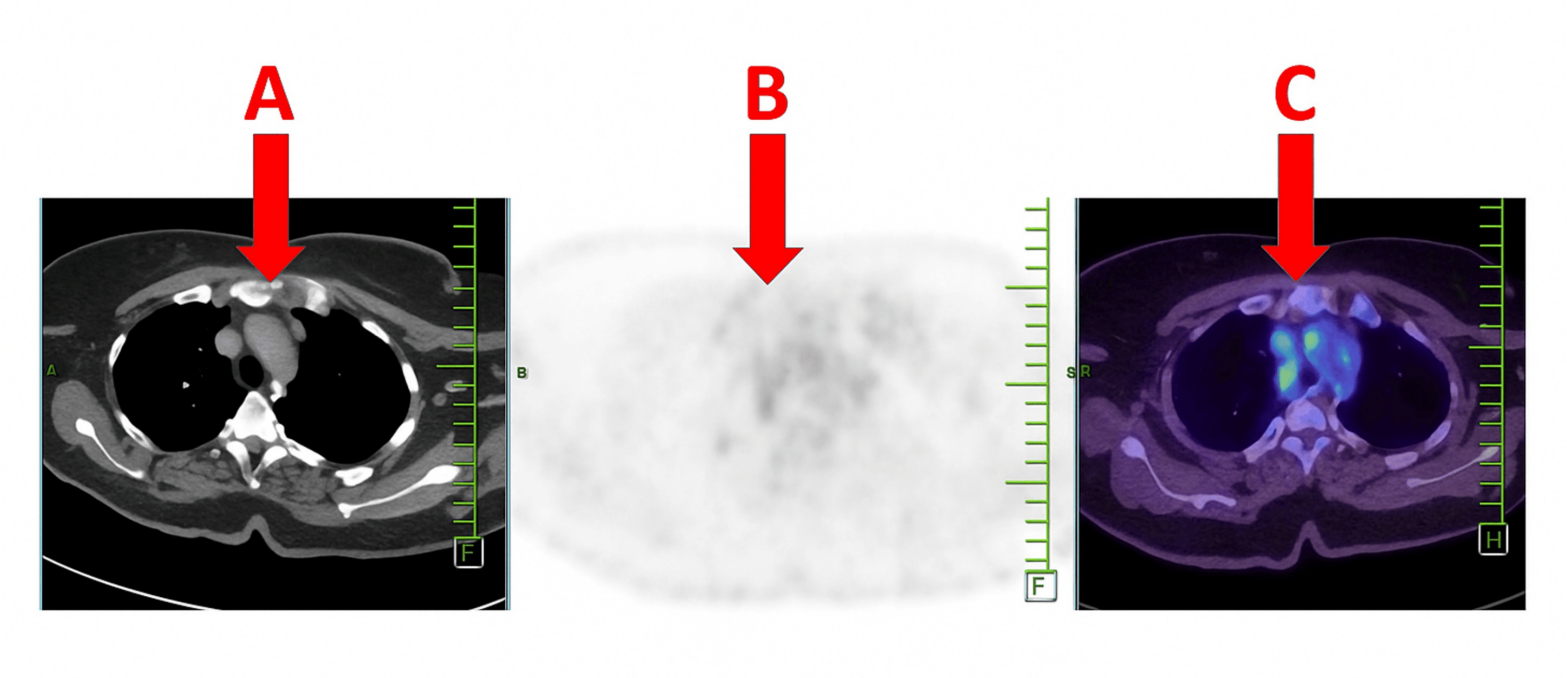
Axial PET-CT images of the thorax. (A) CT showing mediastinal lymph nodes. (B) PET grayscale image. (C) Fused PET-CT highlighting fluorodeoxyglucose-avid mediastinal and hilar lymph nodes (red arrows), consistent with sarcoidosis-related lymphadenopathy.

Baseline investigations are presented in Table 1. Chest radiography revealed bilateral hilar lymphadenopathy (Figure 1). PET-CT demonstrated periportal and mediastinal lymphadenopathy (Figures 2, 3). Endobronchial ultrasound (EBUS) biopsy showed necrotizing granulomatous inflammation.

**Table 1:** Investigations. USG: ultrasonography; LS: lumbosacral; PET-CT: positron emission tomography–computed tomography; FDG: fluorodeoxyglucose; EBUS: endobronchial ultrasound; AFB: acid-fast bacillus; ACE: angiotensin-converting enzyme.

Test	Result	Reference range	Interpretation
Serum calcium	16.7 mg/dL	8.5 – 10.5 mg/dL	Severe hypercalcemia
Serum creatinine	3.7 mg/dL	0.6 – 1.2 mg/dL	Acute kidney injury
Chest X-ray	Bilateral hilar lymphadenopathy	Normal: Clear lung fields, no lymphadenopathy	Suggestive of granulomatous disease
Ultrasonography (USG) neck	Normal	Normal thyroid and parathyroid morphology	No parathyroid abnormality
Vitamin D (total)	Normal	30 – 100 ng/mL	Excludes exogenous cause
X-ray lumbosacral (LS) spine & pelvis	Normal	No abnormal bony lesions	No bony lesions
PET-CT	Periportal and mediastinal lymphadenopathy	Normal: No FDG-avid lymphadenopathy	Systemic involvement
EBUS biopsy	Necrotizing granulomatous inflammation	Normal: No granulomas	Consistent with sarcoidosis
Acid-fast bacillus (AFB) GeneXpert	Negative	Negative	Excludes tuberculosis
Angiotensin-converting enzyme (ACE) levels	92 U/L	8 – 52 U/L (lab-dependent)	Supports sarcoidosis diagnosis

Differential diagnoses considered were tuberculosis, which was excluded based on negative acid-fast bacillus (AFB) testing and absence of constitutional symptoms, malignancy, which was not supported by imaging or pathology, and primary hyperparathyroidism, which was excluded by normal neck ultrasound and vitamin D.

The patient was managed with aggressive hydration consisting of an initial bolus of 0.9% saline at 15 mL/kg IV over two hours, followed by maintenance fluids at 2.5 mL/kg/hour.

Correction of hypercalcemia was done, and intravenous fluids were administered to restore renal perfusion. The patient was started on oral prednisone 40 mg once daily to reduce granulomatous inflammation. Regular monitoring of electrolytes, renal function, and neurological status was done.

Outcome and follow-up

The patient showed marked clinical improvement after initiation of aggressive intravenous hydration and corticosteroid therapy. Calcium decreased to 11.0 mg/dL and creatinine to 1.5 mg/dL. By day three, serum calcium normalized to 9.6 mg/dL and creatinine improved to 1.2 mg/dL, correlating with marked symptomatic improvement.

At two-week outpatient follow-up (day 14), the patient reported sustained improvement in mood, energy levels, and bowel habits. Repeat laboratory investigations showed a serum calcium of 9.4 mg/dL, creatinine of 1.0 mg/dL, and a fall in angiotensin-converting enzyme (ACE) level to 70 U/L. There were no new systemic or neurological symptoms. Prednisone was continued at the same dose for another week, followed by a gradual taper of 5 mg every two weeks under close monitoring.

At one-month follow-up, the patient remained asymptomatic, with normal appetite and no recurrence of constipation or fatigue. Serum calcium and renal parameters remained within the normal range. She had no features of corticosteroid toxicity, and chest imaging showed persistent but non-progressive mediastinal lymphadenopathy.

## Discussion

Sarcoidosis is a multisystem granulomatous disease with variable clinical presentation and outcomes. Pulmonary and lymphatic involvement are most common, but extrapulmonary manifestations can be the initial clue to diagnosis [1,2]. Hypercalcemia occurs in approximately 10-20% of sarcoidosis patients and is mediated by extrarenal conversion of 25-hydroxyvitamin D to 1,25-dihydroxyvitamin D by activated macrophages in granulomatous tissue [3,4]. This results in increased intestinal calcium absorption and reduced renal excretion, which can precipitate acute kidney injury if left untreated [5]. Several case reports and series have described neurocognitive or psychiatric symptoms associated with hypercalcemia secondary to sarcoidosis. In one study, Sharma reported that hypercalcemia may present with depression, confusion, or encephalopathy, often preceding systemic diagnosis [4]. Our patient similarly presented with altered sensorium, initially attributed to a psychiatric disorder, which delayed recognition of the underlying metabolic disturbance. The diagnosis of sarcoidosis requires a combination of compatible clinical and radiological findings, histological confirmation of non-caseating granulomas, and exclusion of alternative causes such as tuberculosis and fungal infections [6]. In endemic areas, distinguishing sarcoidosis from tuberculosis remains particularly challenging; however, negative microbiological studies (including AFB GeneXpert) and necrotizing granulomatous histopathology supported sarcoidosis in our case. The role of serum ACE levels remains controversial. While elevated ACE supports the diagnosis, it lacks sensitivity and specificity [7]. Nevertheless, in conjunction with histology and imaging, it contributed to the diagnostic certainty in this case. PET-CT has emerged as a valuable tool for detecting occult systemic involvement, assessing disease activity, and guiding biopsy sites [8], as demonstrated in our patient. Management of sarcoidosis-related hypercalcemia requires prompt recognition and correction of electrolyte imbalance, aggressive hydration, and corticosteroid therapy [9]. Corticosteroids reduce granulomatous activity and suppress extrarenal vitamin D activation, leading to normalization of calcium levels. Our patient responded well to steroids, with resolution of hypercalcemia and improvement in sensorium.

This case reinforces the need for clinicians to consider sarcoidosis in the differential diagnosis of unexplained hypercalcemia with neurological manifestations. Early diagnosis is crucial to prevent complications such as irreversible renal dysfunction and chronic neurocognitive impairment.

## Conclusions

This case demonstrates that sarcoidosis can rarely present with severe hypercalcemia, causing neuropsychiatric symptoms and acute kidney injury. Hypercalcemia in sarcoidosis results from unregulated calcitriol production by activated macrophages within granulomas and, if unrecognized, can lead to complications such as renal dysfunction and neuropsychiatric manifestations. Careful evaluation with laboratory studies, imaging, and histopathological confirmation is essential to distinguish sarcoidosis from other granulomatous diseases, particularly tuberculosis in endemic areas. In patients with unexplained hypercalcemia and lymphadenopathy, sarcoidosis should be considered after excluding more common causes such as malignancy and tuberculosis. Early recognition and prompt corticosteroid therapy can lead to rapid clinical and biochemical recovery.
